# Plasma Interleukin‐10 Levels and *IL10* Genetic Variants Are Associated With Clinical Outcomes in Patients With Severe COVID‐19 Treated With Corticosteroids

**DOI:** 10.1002/jmv.71201

**Published:** 2026-10-30

**Authors:** Ingrid Fricke‐Galindo, Francisco M. Ortega‐Villafuerte, Gloria Pérez‐Rubio, Ivette Buendía‐Roldán, Julián Ramírez‐Bello, Azael A. Castillo Morales, Leslie Chávez‐Galán, Rafael de Jesús Hernández‐Zenteno, Espiridión Ramos‐Martínez, Salvador García‐Carmona, Daniela Josefina Cataneo‐Piña, Nora Edith Bautista‐Félix, Ramcés Falfán‐Valencia

**Affiliations:** ^1^ Pneumogenomics Laboratory Instituto Nacional de Enfermedades Respiratorias Ismael Cosío Villegas Mexico City Mexico; ^2^ Escuela Superior de Medicina Instituto Politécnico Nacional Mexico City Mexico; ^3^ Translational Research Laboratory on Aging and Pulmonary Fibrosis Instituto Nacional de Enfermedades Respiratorias Ismael Cosío Villegas Mexico City Mexico; ^4^ Subdirección de Investigación Clínica Instituto Nacional de Cardiología Ignacio Chávez México City Mexico; ^5^ Facultad de Ciencias Químico‐Biológicas Universidad Autónoma de Sinaloa, Culiacán Rosales Sinaloa Mexico; ^6^ Laboratory of Integrative Immunology Instituto Nacional de Enfermedades Respiratorias Ismael Cosío Villegas Mexico City Mexico; ^7^ Tobacco Smoking and COPD Research Department Instituto Nacional de Enfermedades Respiratorias Ismael Cosio Villegas Mexico City Mexico; ^8^ Unidad de Medicina Experimental, Facultad de Medicina Universidad Nacional Autónoma de México Ciudad de México Mexico; ^9^ Department of Geriatrics and Palliative Care Instituto Nacional de Enfermedades Respiratorias Ismael Cosio Villegas Mexico City Mexico; ^10^ Red Mexicana de Investigación Clínica en Enfermedades Emergentes Infecciosas (LaRed) Instituto Nacional de Enfermedades Respiratorias Ismael Cosio Villegas Mexico City Mexico

**Keywords:** corticosteroids, COVID‐19, dexamethasone, IL‐10, single‐nucleotide variant

## Abstract

We aimed to evaluate the association between IL10 variants and plasma IL‐10 levels and clinical outcomes in patients with severe COVID‐19 treated with corticosteroids. We studied 944 patients with severe COVID‐19 who were treated with corticosteroids. The clinical outcomes evaluated were in‐hospital mortality and the requirement of invasive mechanical ventilation (IMV). The variants *IL10* rs1800872, rs1554286, rs3024505, and rs3122605 were determined using TaqMan SNP assays, and IL‐10 levels were measured by ELISA at the start of corticosteroid treatment (T1) and after 8 days (T2). The rs1800872‐TT (*p* = 0.035, OR = 1.55, 95% CI = 1.03–2.33) and the rs1554286‐AA genotypes were associated with IMV (*p*_adj_ = 0.036, OR = 2.42, 95% CI = 1.12–5.95). IL‐10 levels were higher at T1 than at T2. At T2, IL‐10 levels were higher in non‐survivors (15.9 vs. 7.0 pg/mL, *p* < 0.001) and in patients requiring IMV (9.9 vs. 5.5 pg/mL, *p* < 0.001). Among non‐survivors, IL‐10 plasma levels did not decrease at T2. The alluvial plot revealed that 51.9% of patients in the highest IL‐10 tertile at T1 remained in the highest tertile at T2, with a mortality rate of 50%. *IL10* rs1800872 and rs1554286 contribute to the risk of requiring IMV. Elevated IL‐10 levels after corticosteroid treatment were linked to in‐hospital mortality and IMV.

## 1 Background

The coronavirus disease 2019 (COVID‐19) pandemic was a global public health concern due to its spread, clinical variability, and potential for mortality [1]. The respiratory virus SARS‐CoV‐2 causes this disease, and the clinical presentation can vary depending on host factors and the pathogenesis of different virus variants [2]. About 32% of infected subjects were asymptomatic, in whom an inflammatory response was not detectable [3]. Meanwhile, most of the symptomatic cases (80%) present mild to moderate disease; 15% develop severe disease, requiring oxygen support; and 5% establish a critical disease with complications such as respiratory failure, acute respiratory distress syndrome (ARDS), sepsis and septic shock, thromboembolism, and/or multi‐organ failure [4, 5].

ARDS is frequently associated with sepsis, and both conditions are characterized by excessive inflammation [6]. The SARS‐CoV‐2 entry into the organism triggers different inflammatory responses through cytokine and chemokine responses, the expression of antiviral factors, pulmonary cell infiltration, increased vascular permeability, and lymphopenia [7]. A dysregulated inflammatory response can cause irreparable cellular damage and lung injury. The overproduction of pro‐inflammatory cytokines has been associated with severe forms of COVID‐19 and increased mortality risk [8]. Mitigating inflammatory organ injury in viral pneumonia was a determinant of improvement in patients with COVID‐19. In this context, the RECOVERY Collaborative Group conducted a controlled, open‐label trial comparing treatments in patients hospitalized with COVID‐19. This group reported that the use of dexamethasone, a widely used corticosteroid, decreased 28‐day mortality among patients hospitalized with COVID‐19 who received either invasive mechanical ventilation (IMV) or oxygen alone, but not among those receiving no respiratory support [9].

It has been proposed that corticosteroids exert anti‐inflammatory effects in ARDS by attenuating neutrophil recruitment and reducing the transcription of proinflammatory mediators. This could mitigate alveolar epithelial and endothelial injury, reduce alveolar edema fluid accumulation, improve prognosis, and reduce mortality in patients with ARDS. However, the efficacy of corticosteroids in this syndrome depends on the timing and duration of treatment, as well as the cause of ARDS [10]. In addition, the efficacy and safety of these drugs exhibit substantial interindividual variability, suggesting the potential for personalized medicine to optimize corticosteroid therapy [11].

Host genetic variability may contribute to heterogeneity in inflammatory responses and treatment outcomes among patients with severe COVID‐19. A pilot study of hospitalized patients found an association between variants in the glucocorticoid pathway (*CYP3A4* rs35599367, *NR3C1* rs6198, rs33388, and *GSTP1* rs1695) and response to dexamethasone and/or methylprednisolone across different clinical outcomes [12]. A recent GWAS reported that the genetic determinants of treatment response in patients with COVID‐19 include not only drug pharmacokinetics and pharmacodynamics, but also genes involved in the innate immune system, programmed cell death, and host susceptibility to viral infection pathways, which could modulate drug responses [13]. In this context, interleukin 10 (IL‐10) has been proposed as a biomarker and incorporated into a laboratory‐based nomogram to predict clinical outcomes in patients with severe COVID‐19 treated with corticosteroids. The authors reported that elevated IL‐10 levels were associated with corticosteroid resistance and, along with ferritin and diabetes, could predict treatment failure [14].

IL‐10 is an essential cytokine, mainly produced by macrophages, that suppresses the inflammatory response. It blocks antigen presentation and the release and activity of proinflammatory cytokines, such as IL‐6, TNF‐α, and IL‐1β [15]. The increase in IL‐10 production in response to glucocorticoids has been linked to the drugs' suppressive effects [16, 17] and to the viral infection process [18]. Controversial results have been reported regarding its association with COVID‐19 severity [19, 20].

Thus, the scientific literature recommends administering corticosteroids to patients with severe COVID‐19 to improve clinical outcomes, but their efficacy and safety vary. Additional research is needed to fully understand the role of corticosteroids in the treatment of ARDS and to determine the genetic and non‐genetic factors that contribute to treatment success [6]. Therefore, we aimed to evaluate the association between *IL10* variants, IL‐10 plasma levels, and clinical outcomes in patients with severe COVID‐19 treated with corticosteroids.

## 2 Methods

### 2.1 Participants

This was an observational, retrospective, and longitudinal study. We consecutively recruited 944 patients with severe COVID‐19 who were hospitalized (July 2020–January 2022) at the Instituto Nacional de Enfermedades Respiratorias Ismael Cosío Villegas, a third‐level reference center for respiratory diseases in Mexico. The study was conducted in accordance with the Declaration of Helsinki and received approval from the local Committees for Research, Biosecurity, and Ethics in Research (approval code B03‐24). Written informed consent was obtained from each participant after they were invited and informed of the study's purpose and scope.

We included only patients ≥ 18 years old with confirmed COVID‐19 by RT‐PCR who received corticosteroids (approximately 70% dexamethasone alone, and 30% methylprednisolone, prednisolone, hydrocortisone, or in combination with inhaled corticosteroids) during their hospital stay. Patients with incomplete information or lacking blood samples were excluded. Patients had severe COVID‐19 presenting with dyspnea, a respiratory rate of ≥ 30 breaths per minute, blood oxygen saturation ≤ 90%, and/or PaO_2_/FiO_2_ ≤ 300. The response to corticosteroids was evaluated by in‐hospital mortality and the requirement of IMV at the end of the hospital stay. We excluded patients with unknown clinical outcomes and those who declined the IMV procedure.

### 2.2 Genotyping

DNA was isolated from blood samples collected in EDTA‐containing tubes. Samples were centrifuged to separate plasma from the blood cells. The plasma was stored at −80°C until use. DNA was isolated using the Blood DNA Preparation Solution Kit (Jena Bioscience, Jena, Thuringia, Germany). After a literature review of the *IL10* variants relevant to respiratory diseases and/or immune disorders, we selected the rs1800872, rs1554286, rs3024505, and rs3122605 for this study. These variants were genotyped using TaqMan SNP genotyping assays (C_1747363_10, C_8828812_10, C_15983681_20, and C_31404381_10, respectively) on a StepOnePlus System (Applied Biosystems, Foster City, CA, USA) according to the supplier's instructions.

### 2.3 Determination of IL‐10 Plasma Levels by ELISA

In a subgroup of 160 patients, plasma IL‐10 levels were measured at two time points during corticosteroid treatment. Patients were included if the sample storage date matched the start of corticosteroid treatment (± 1 day, T1) and at a mean of 8 days later (± 1 day, T2). Samples had to meet quality control criteria for ELISA analysis, and the plasma volume had to be sufficient for IL‐10 determination. Most patients (*n* = 114, 71.25%) were treated with dexamethasone; 13.75% received dexamethasone at the start and then switched to methylprednisolone/prednisolone (*n* = 22); and 15% received dexamethasone plus an inhaled corticosteroid (budesonide, fluticasone) (*n* = 24). The standard dosage of dexamethasone was 6 mg once a day, intravenously, for 9 days.

Plasma IL‐10 levels were determined using the Human IL‐10 ELISA MAX Deluxe Set (BioLegend, San Diego, CA, USA) according to the manufacturer's instructions. The standard curve ranged from 0.97 to 125 pg/mL. The absorbance was read at 450 nm. All samples were assessed in duplicate, and the mean value per well was reported in pg/mL.

### 2.4 Statistical Analysis

The genetic association study was performed in PLINK v1.07 [21], including logistic regression for covariate adjustment. The covariates included in the models corresponded to the clinical and demographic variables that differed among the comparison groups (in‐hospital mortality and the requirement for IMV at the end of the hospital stay). The allele and genotype frequencies are expressed as absolute numbers and proportions. Haploview v.4.2 [22] was used to assess linkage disequilibrium (LD). The statistical analyses were carried out in RStudio v.4.5.1 [23]. The categorical variables are presented as absolute numbers and percentages, while the continuous data are reported as medians (interquartile range, Q1–Q3). The normality of the data distribution was evaluated using the Kolmogorov‐Smirnov test, as applicable. The chi‐square test was used to evaluate the independence of two nominal variables, and the Mann–Whitney *U*‐test was used to compare continuous data between groups. The Spearman rank correlation test evaluated the strength and direction of association between the IL‐10 plasma levels and continuous variables. A generalized linear regression model was used to evaluate the effects of potential confounding variables on IL‐10 plasma levels and clinical outcomes. The variance inflation factor (VIF) and tolerance were assessed for all independent variables to evaluate collinearity in the adjusted model; VIF values < 3.0 and tolerance > 0.10 indicate the absence of significant multicollinearity among the predictors. The statistical significance was set at *p* < 0.05.

## 3 Results

### 3.1 Clinical and Demographic Data of Patients With Severe COVID‐19

The group of 944 patients with severe COVID‐19 treated with corticosteroids was classified according to in‐hospital mortality and IMV requirement. Complete discharge information was available for 924 patients, of whom 25.4% died during their hospital stay. Meanwhile, all recruited patients (*n* = 944) were included in the analysis of IMV requirement after corticosteroid use, of whom more than half (69.2%) required IMV.

Table 1 presents clinical and demographic data for patients classified by in‐hospital mortality and IMV requirement. Older age and the presence of concomitant heart diseases were found to be associated with the risk of both clinical outcomes. Likewise, lower PaO_2_/FiO_2_ levels at admission, coinfections, and a longer hospital stay were observed in the non‐survivors and IMV groups.

**Table 1:** Clinical and demographic data of patients with severe COVID‐19 treated with corticosteroids.

Variable	Non‐survivors (*n* = 235)	Survivors (*n* = 689)	*p*	IMV (*n* = 653)	Non‐IMV (*n* = 291)	*p*
Age, years	65 [53–73]	55 [45–65]	**< 0.001**	59 [49–69]	54 [44–64]	**< 0.001**
BMI, kg/m^2^	27.8 [24.9–31.2]	29.1 [26.0–33.3]	**0.002**	28.7 [25.7–32.7]	28.3 [25.5–32.5]	0.166
Sex, male, *n* (%)	163 (69.4)	438 (63.6)	0.114	438 (67.1)	178 (61.2)	0.167
Comorbidities, *n* (%)						
Tobacco‐smoking	76 (32.3)	203 (29.5)	0.939	201 (30.8)	91 (31.3)	0.939
T2DM	67 (28.5)	188 (27.3)	0.736	178 (27.3)	86 (29.5)	0.481
SAH	91 (38.7)	230 (33.4)	0.154	242 (37.1)	90 (30.9)	0.065
CRD	21 (8.9)	48 (7.0)	0.319	38 (5.8)	33 (11.3)	**0.004**
Heart disease	17 (7.2)	17 (2.5)	**0.002**	30 (4.6)	5 (1.7)	**0.038**
Clinical data at admission						
Days since symptoms onset	9 [7–14]	9 [7–12]	0.475	9 [7–13]	9 [7–12]	0.938
PaO_2_/FiO_2_	147 [115–176]	163 [125–211]	**< 0.001**	147 [116–180]	197 [144–242]	**< 0.001**
COVID‐19 vaccination, *n* (%)	41 (17.4)	116 (16.8)	0.840	131 (20.1)	38 (13.1)	**0.010**
Clinical outcomes						
Coinfection, *n* (%)	120 (51.1)	240 (34.8)	**< 0.001**	349 (53.4)	28 (9.6)	**< 0.001**
Hospital stay, days	21 [14–30]	16 [10–27]	**< 0.001**	22 [15–34]	10 [7–13]	**< 0.001**
IMV, *n* (%)	226 (96.2)	406 (58.9)	**< 0.001**	653 (100)	0 (0)	NA
IMV days	20 [13–30]	8 [0.00–16]	**< 0.001**	16 [10–28]	0	NA
Mortality, *n* (%)	235 (100)	0 (0)	NA	226 (34.6)	9 (3.1)	**< 0.001**

*Note:* Continuous data are presented as median [interquartile range, Q1–Q3]. Categorical data are presented in absolute numbers (%). Exact Fisher's and Mann–Whitney *U*‐tests were used for comparisons. Text in bold denotes a significant *p* value.

Abbreviations: BMI, body mass index; CRD, chronic respiratory disease; IMV, invasive mechanical ventilation; SAH, systemic arterial hypertension; T2DM, Type 2 diabetes mellitus.

The median body mass index (BMI) was higher among survivors than non‐survivors (29.1 vs. 27.8 kg/m^2^, *p* = 0.002), although no differences were observed between the IMV requirement groups. Male frequency was not a relevant factor for in‐hospital mortality or IMV requirement. We also did not observe an effect of COVID‐19 vaccination on the decrease in mortality or severity. Instead, the IMV group had a higher proportion of patients who received COVID‐19 vaccination than the non‐IMV group (20.1% vs. 13.1%, *p* = 0.010).

Regarding the comorbidities, the frequency of chronic respiratory disease (CRD) was higher among non‐IMV than in the IMV group. In contrast, the frequencies of tobacco‐smoking subjects, Type 2 diabetes mellitus (T2DM), and systemic arterial hypertension (SAH) were not statistically significantly different between the comparison groups.

Most patients in the non‐survivors group (96.2%) received IMV for a longer duration than survivors (20 days vs. 8 days, *p* < 0.001). Meanwhile, the mortality rate was 10‐fold higher in the IMV group than in the non‐IMV group (34.6% vs. 3.1%, *p* < 0.001). Patients requiring IMV were more likely to develop coinfections during hospitalization (53.4% vs. 9.6%, *p* < 0.001).

We also evaluated whether corticosteroid treatment affected the clinical outcomes. Most patients received dexamethasone alone in each group (non‐survivors 64.6%, survivors 75.8%, IMV 70.3%, and non‐IMV 77.6%), and the remaining patients received an additional corticosteroid and/or inhaled (35.4%, 24.2%, 29.7%, and 22.4%, respectively). The frequencies did not differ among the comparison groups. Thus, the corticosteroid regimen was not associated with in‐hospital mortality (*p* = 0.100) or IMV requirement (*p* = 0.312).

### 3.2 IL10 Genetic Variants Are Associated With the Requirement of IMV in Patients With COVID‐19 Treated With Corticosteroids

We evaluated the association of the *IL10* rs3024505, rs3122605, rs1554286, and rs1800872 variants with in‐hospital mortality and the requirement for IMV in patients with COVID‐19 treated with corticosteroids. The allele and genotype frequencies of the variants were similar between the non‐survivor and survivor groups; thus, none of the studied variants were associated with in‐hospital mortality in patients with COVID‐19 receiving corticosteroid treatment (Table S1).

On the other hand, rs1800872 was associated with the need for IMV among patients with COVID‐19 who received corticosteroids. Specifically, patients with the TT genotype under the recessive model had a higher risk of IMV requirement than carriers of GG + GT genotypes (Table 2, *p* = 0.035, OR = 1.55, 95% CI = 1.03–2.33). The remaining variants were not associated with the IMV requirement, although a tendency was observed for the AA genotype to be more frequent under the recessive model at rs1554286 (Table 2) compared with the GG + GA genotypes. We also performed logistic regression to adjust for covariates (age, BMI, heart disease, PaO_2_/FiO_2_ at admission, and coinfection). We performed a multicollinearity test and found no correlation among the independent variables (Table S2). The statistical significance of rs1800872 was lost; meanwhile, the difference observed for rs1554286 under the recessive model was now statistically significant (*p*_adj_ = 0.036, OR = 2.42, 95% CI = 1.12–5.95). However, the frequencies of the other three variants did not differ significantly between the IMV and non‐IMV groups.

**Table 2:** Genetic association study of *IL10* variants with invasive mechanical ventilation requirement among patients with COVID‐19 treated with corticosteroids.

*IL10* allele/genotypes	IMV (*n* = 653)^a^	Non‐IMV (*n* = 291)^a^	*p*	Adjusted *p* b
rs3024505	*n* = 621	*n* = 270		
G	1184 (0.953)	506 (0.937)	0.154	
A	58 (0.047)	34(0.063)		
GG	563 (0.907)	236 (0.874)	0.142	
GA	58 (0.093)	34 (0.126)		0.990
AA	0	0		
rs3122605	*n* = 417	*n* = 194		
A	807 (0.968)	370 (0.954)	0.226	
G	27 (0.032)	18 (0.046)		
AA	390 (0.935)	176 (0.907)	0.217	
AG	27 (0.065)	18 (0.093)		0.846
GG	0	0		
rs1554286	*n* = 621	*n* = 268		
G	610 (0.491)	285 (0.532)	0.116	0.999
A	632 (0.509)	251 (0.468)		
GG	148 (0.238)	71 (0.265)	0.207	
GA	314 (0.506)	143 (0.534)		0.998
AA	159 (0.256)	54 (0.201)		0.064
Dominant/recessive model				
GA + AA	473 (0.762)	197 (0.735)	0.398	0.500
GG	148 (0.238)	71 (0.265)		
AA	159 (0.256)	54 (0.201)	0.080	**0.036**
GG + GA	462 (0.744)	214 (0.799)		
rs1800872	*n* = 613	*n* = 267		
G	681 (0.555)	318 (0.596)	0.119	0.999
T	545 (0.445)	216 (0.404)		
GG	184 (0.300)	86 (0.322)	0.109	
GT	313 (0.511)	146 (0.547)		0.278
TT	116 (0.189)	35 (0.131)		0.492
Dominant/recessive model				
GT + TT	429 (0.700)	181 (0.678)	0.517	0.514
GG	184 (0.300)	86 (0.322)		
TT	116 (0.189)	35 (0.131)	**0.035**	0.184
GG + GT	497 (0.811)	232 (0.869)		

*Note:* The alleles and genotypes are presented as absolute numbers and proportions between brackets. Text in bold denotes a significant *p* value.

^a^ Genotyping was performed according to sample availability; thus, the number of genotyped subjects is specified for each case.

^b^ Logistic regression model for the adjustment of covariates: age, body mass index, heart disease, PaO_2_/FiO_2_ at admission, and coinfection.

The haplotype analysis indicated moderate LD between the two associated variants for IMV (rs1554286 and rs1800872; *r* ^2^ = 63, *D*′ = 91). Meanwhile, the rs3024505 variant showed a low LD with rs3122605 (*r* ^2^ = 37, *D*′ = 75) (Figure S1). Nevertheless, the haplotype association study did not show any significant difference in haplotype frequency between the IMV and non‐IMV groups (Table S3). A marginal *p* value (0.060) was observed for the haplotype that includes the common alleles at rs1554286 and rs1800872 (GG), which is more frequent in the non‐IMV group. The other three combinations (AT, AG, and GT) did not show any significant difference either (Table S3).

### 3.3 The Increase in Plasma IL‐10 Levels Is Associated With In‐Hospital Mortality and the Need for IMV Among COVID‐19 Patients Treated With Corticosteroids

In a subgroup of patients (*n* = 160), we measured plasma IL‐10 levels at two time points during corticosteroid treatment: at the start of treatment (T1) and after a mean of 8 days (T2). Patients in this subgroup differed from the rest of the cohort, primarily because those selected for IL‐10 plasma level measurement were slightly older and exhibited more severe disease symptoms, including fewer days since symptom onset, more frequent use of IMV, and longer hospital stays (see Table S4). Despite these differences, the subgroup was classified based on clinical outcomes, and the observed variations in clinical and demographic features were included as covariates in each association analysis.

The clinical and demographic characteristics of this subgroup are presented in Table 3; note that, in this subgroup, older age, SAH, heart disease, lower PaO_2_/FiO_2_ levels at admission, and coinfections were more frequent in the non‐survivor group. Meanwhile, more days since symptom onset, lower PaO2/FiO2 levels at admission, and a lower COVID‐19 vaccination rate were observed in the IMV group compared with the non‐IMV group.

**Table 3:** Clinical and demographic variables of the subgroup of patients included in the analysis of IL‐10 plasma levels (*n* = 160).

Variable	Non‐survivors (*n* = 48)	Survivors (*n* = 112)	*p*	IMV (*n* = 123)	Non‐IMV (*n* = 37)	*p*
Demographic						
Age, years	66 [57–73]	57 [50–65]	**0.002**	61 [51–70]	57 [52–65]	0.443
BMI, kg/m^2^	28.0 [25.4–33.4]	29.3 [26.2–33.6]	0.502	29.0 [26.2–33.7]	27.6 [25.2–33.1]	0.290
Sex, male, *n* (%)	35 (73)	75 (67)	0.577	89 (72.4)	21 (56.8)	0.104
Comorbidities, *n* (%)						
Tobacco smoking	14 (29.2)	34 (30.3)	1.000	37 (30.1)	11 (29.7)	1.000
T2DM	19 (39.6)	32 (28.6)	0.197	38 (30.9)	13 (35.1)	0.689
SAH	26 (54.2)	38 (33.9)	**0.021**	52 (42.3)	12 (32.4)	0.341
CRD	3 (6.2)	5 (4.4)	0.697	4 (3.3)	4 (10.8)	0.084
Heart disease	6 (12.5)	1 (0.9)	**0.003**	7 (5.7)	0 (0)	0.354
Clinical data at admission						
Days since symptoms onset	8 (6–12)	9 (7–11)	0.511	9 (7–13)	7 (6–10)	**0.045**
PaO_2_/FiO_2_	151 (131–173)	171 (134–213)	**0.048**	155 (129–181)	201 (161–243)	**< 0.001**
COVID‐19 vaccine, *n* (%)	3 (6.2)	11 (9.8)	0.556	8 (6.5)	6 (16.2)	**0.047**
Clinical outcomes						
Coinfection, *n* (%)	22 (45.8)	40 (35.7)	**0.004**	57 (46.3)	5 (13.5)	0.704
Hospital stay, days	21 (15–27)	19 (14–25)	0.237	21 (16–28)	13 (12–20)	**< 0.001**
IMV, *n* (%)	47 (97.9)	76 (67.8)	**< 0.001**	123 (100)	0 (0)	NA
IL‐10 levels T1, pg/mL	17.0 (7.6–30.7)	12.5 (7.4–20.6)	0.112	14.3 (7.7–25.0)	11.2 (7.1–19.3)	0.064
IL‐10 levels T2, pg/mL	15.9 (7.8–26.5)	6.9 (4.5–12.5)	**< 0.001**	9.9 (5.5–20.0)	5.5 (4.1–8.8)	**< 0.001**

*Note:* Continuous data are presented as median [interquartile range, Q1–Q3]. Categorical data are presented in absolute numbers (%). Exact Fisher's and Mann–Whitney *U*‐tests were used for comparisons. Text in bold denotes a significant *p* value.

Abbreviations: BMI, body mass index; CRD, chronic respiratory diseases; IMV, invasive mechanical ventilation; SAH, systemic arterial hypertension; T1, IL‐10 plasma levels at the start of corticosteroid treatment; T2, IL‐10 plasma levels at a mean of 8 days of corticosteroid treatment; T2DM, type 2 diabetes mellitus.

Overall, IL‐10 plasma levels at T1 were higher than at T2. At T1, IL‐10 levels showed no significant difference between non‐survivors and survivors (17.1 pg/mL vs. 12.5 pg/mL, *p* = 0.112), but a trend was observed for the IMV requirement (14.3 pg/mL vs. 11.2 pg/mL, *p* = 0.064; Figure 1). However, at T2, IL‐10 plasma levels were significantly higher in non‐survivors (15.9 pg/mL vs. 7.0 pg/mL, *p* < 0.001) and in patients who required IMV during hospitalization (9.9 pg/mL vs. 5.5 pg/mL, *p* < 0.001; Figure 1).

**Figure 1:**
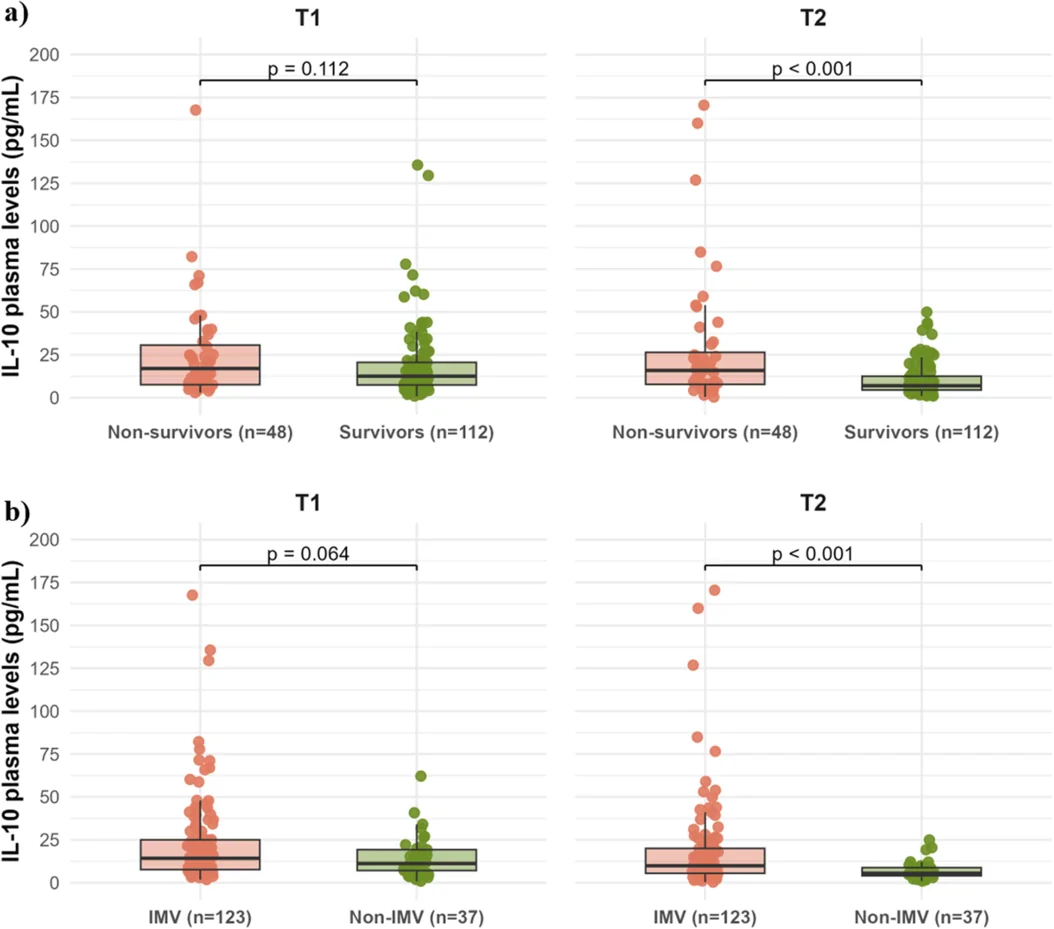
Box plots of plasma IL‐10 levels at two time points during corticosteroid treatment in patients with severe COVID‐19. (a) Differences in IL‐10 plasma levels in patients grouped by in‐hospital mortality and at two times during treatment. (b) Differences in IL‐10 plasma levels in patients grouped by IMV requirement and at two times during treatment. Mann–Whitney *U*‐test.

We fit a logistic regression model to assess the effect of IL‐10 plasma levels on clinical outcome, adjusting for covariates that differed significantly between groups (see Tables S5 and S6). At T2, IL‐10 plasma levels were associated with in‐hospital mortality (*p*_adj_ = 0.002), regardless of age, SAH, PaO_2_/FiO_2_ at admission, or coinfection. However, the significance observed for IL‐10 plasma levels by IMV requirement at T2 was lost after adjustment for days since symptom onset, PaO_2_/FiO_2_ at admission, and COVID‐19 vaccination history (*p*_adj_ = 0.067).

We performed a Wilcoxon signed‐rank test to assess differences in plasma IL‐10 levels during corticosteroid treatment. Among survivors, we found that IL‐10 levels decreased significantly at T2 compared with T1 (12.5 pg/mL vs. 7.0 pg/mL, *p* < 0.001). However, in the non‐survivors group, IL‐10 levels remained high during corticosteroid treatment (17.0 pg/mL vs. 15.9 pg/mL, *p* = 0.571). Additionally, IL‐10 levels differed between T1 and T2 in both the IMV and non‐IMV groups (14.3 pg/mL vs. 9.9 pg/mL, *p* = 0.004, and 11.2 pg/mL vs. 5.5 pg/mL, *p* < 0.001, respectively). Notably, patients in the IMV group showed higher IL‐10 levels than those in the non‐IMV group (Figure 2).

**Figure 2:**
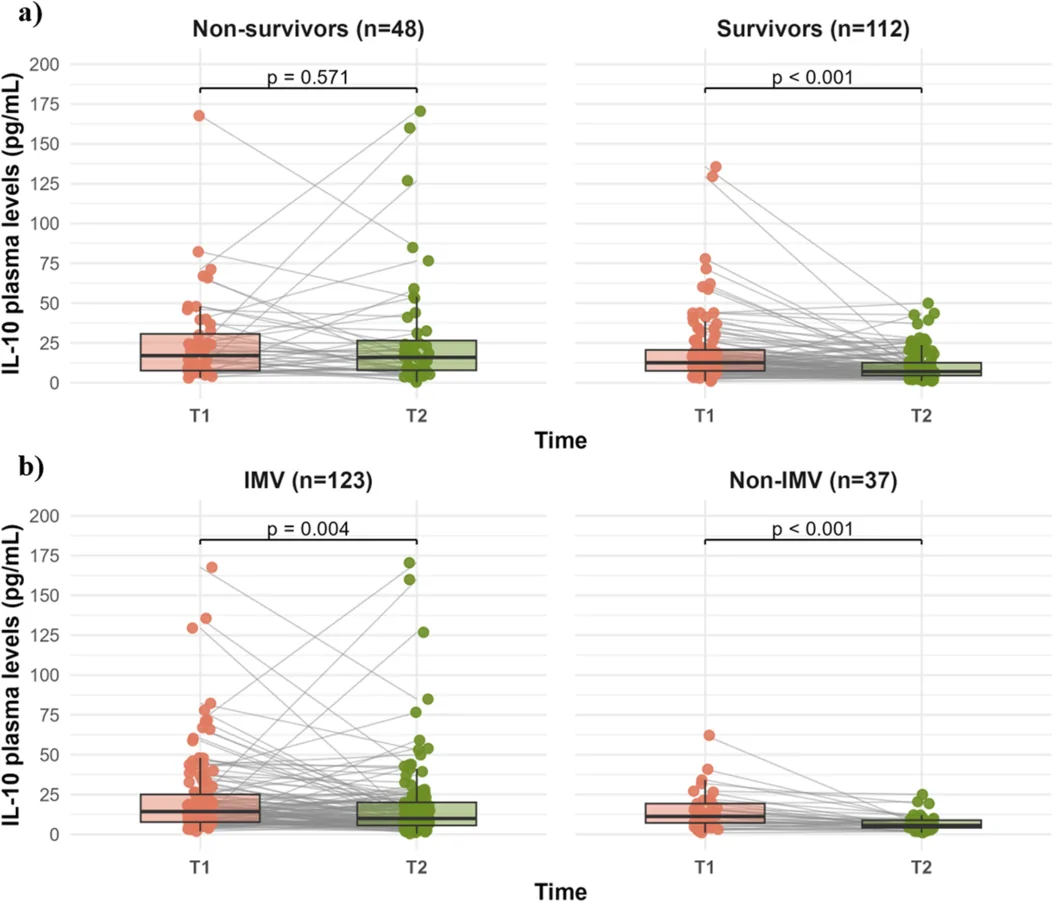
IL‐10 plasma levels at two points during corticosteroid treatment in patients with severe COVID‐19 (T1, at the start of corticosteroid treatment; T2, after a mean of 8 days of corticosteroid treatment). (a) Comparison of IL‐10 plasma levels at these two points in patients grouped by in‐hospital mortality. (b) Comparison of IL‐10 plasma levels at these two points in patients grouped by the need for IMV. Wilcoxon signed‐rank test.

We then analyzed whether the *IL10* variants studied were associated with IL‐10 plasma levels in COVID‐19 patients treated with corticosteroids. However, we found no significant differences in plasma IL‐10 levels across genotypes at the *IL10* rs3024505, rs3122605, and rs1800872 variants (Figures S2 and S3). A difference (*p* = 0.046) was seen in IL‐10 plasma levels based on the genotypes of rs1554286 in the non‐IMV group. Still, this significance did not hold after multiple comparison tests (Figure S3).

Likewise, we inquired about the effects of clinical and demographic variables on IL‐10 plasma levels. At T1, we found higher IL‐10 plasma levels among patients with SAH (16.4 pg/mL vs. 12.0 pg/mL, *p* = 0.027) than those without the disease, and a weak correlation of the IL‐10 levels with age (*ρ* = 0.201, *p* = 0.011) and a negative correlation with days since symptom onset (*ρ* = −0.190, *p* = 0.016). Meanwhile, IL‐10 plasma levels at T2 were only discretely correlated with hospital stay days (*ρ* = −0.256, *p* < 0.001). Sex, tobacco smoking, T2DM, CRD, heart disease, coinfection, COVID‐19 vaccination history, BMI, and PaO_2_/FiO_2_ levels at admission were not related to IL‐10 plasma levels at T1 or T2 (Tables S5 and S6).

Additionally, to integrate and visualize the flow of IL‐10 plasma levels and their connection to clinical outcomes in COVID‐19 patients treated with corticosteroids, we created an alluvial plot (Figure 3). Plasma IL‐10 levels were categorized into tertiles at T1 and T2. Most patients (83%) in the lowest tertile (0.93 to < 8.71 pg/mL) at T1 remained in the lowest and middle tertiles, while 17% changed to the highest tertile. The middle tertile of T1 (8.71 to < 19.85 pg/mL) showed heterogeneous behavior, with about one‐third of patients transitioning to each of the other tertiles at T2. Meanwhile, 51.9% of patients in the highest tertile at T1 (≥ 19.86 pg/mL) remained in the highest tertile at T2 (≥ 13.10 pg/mL; Table S7). At T2, patients in the two lowest tertiles showed a similar pattern. All patients in the lowest (0.38 to < 5.88 pg/mL) and middle (5.88 to < 13.10 pg/mL) tertiles who did not require IMV survived, with mortality rates around 20% in these tertiles. Meanwhile, most patients (94.4%) in the highest IL‐10 plasma level tertile required IMV, and in‐hospital mortality increased to 50% (Table S8).

**Figure 3:**
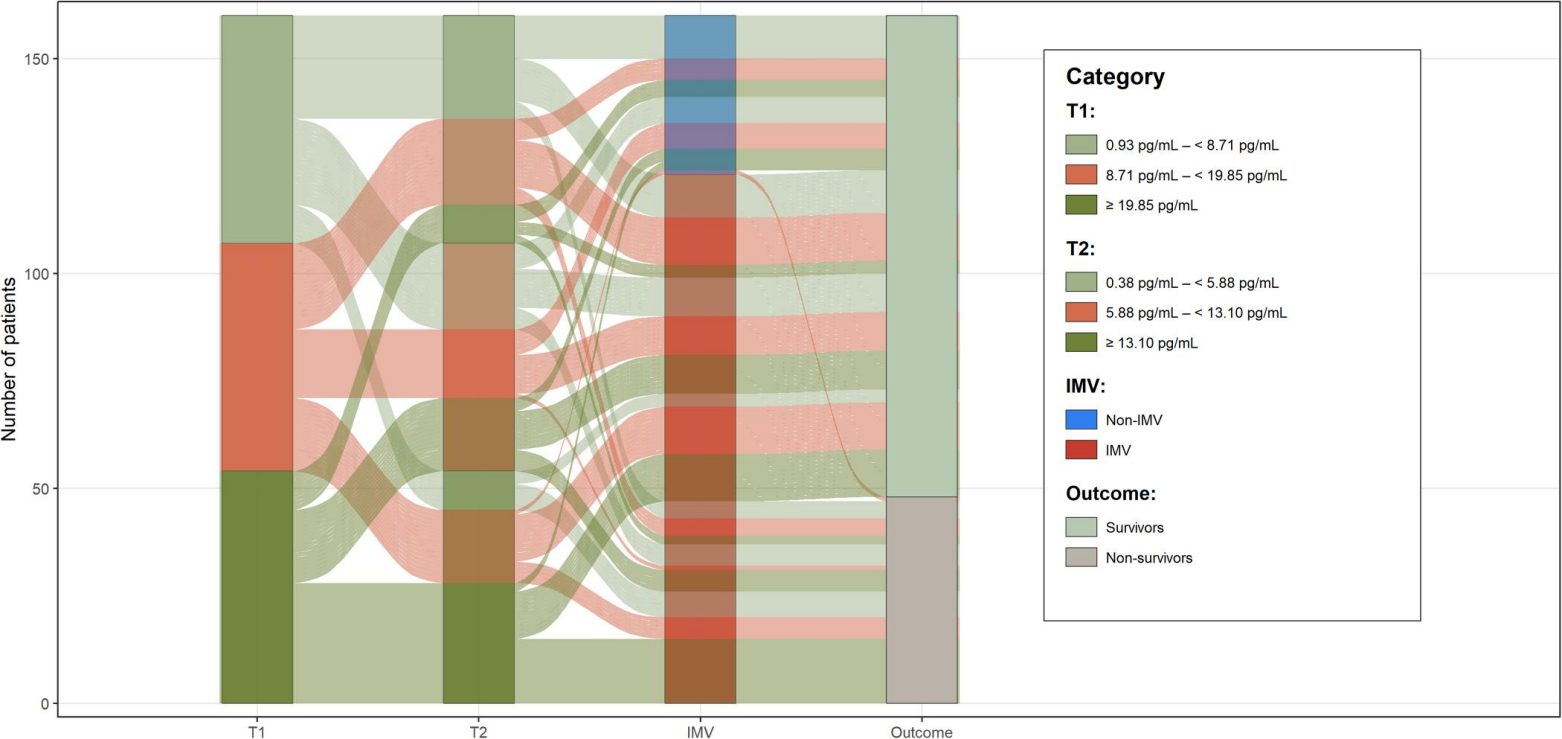
Alluvial plot showing the transition of patients with COVID‐19 according to their IL‐10 plasma levels at two time points during corticosteroid treatment, and their relation to IMV requirement and in‐hospital mortality. IMV, invasive mechanical ventilation; T1, at the onset of corticosteroid treatment; T2, after a mean of 8 days of corticosteroid treatment. IL‐10 plasma levels at T1 and T2 were categorized according to tertiles.

In sum, the frequency of IMV requirement (64.2%, 71.7%, and 94.4%) and in‐hospital mortality (18.9%, 20.8%, and 50.0%) increased as IL‐10 plasma levels increased across the tertiles at T2 (Table S9).

### 3.4 Effect of Timing of Corticosteroid Initiation and the Type of Corticosteroid Treatment on the IL‐10 Plasma Levels and Clinical Outcomes in Patients With COVID‐19

In a follow‐up analysis, including subjects with available data, we assessed the impact of the timing of corticosteroid initiation and the use of other corticosteroids on plasma IL‐10 levels. Based on when corticosteroids were initiated, we divided patients into two groups: early (initiated within ≤ 7 days of symptom onset) and late (> 7 days after symptom onset). Using the Wilcoxon signed‐rank test, we found that IL‐10 plasma concentrations decreased significantly from T1 to T2 in both groups (early: *n* = 49, *p* = 0.008; late: *n* = 104, *p* < 0.001; Figure 4a). However, the early group had higher IL‐10 plasma levels than the late group at T1 (19.55 pg/mL [9.91–26.51 pg/mL] vs. 11.72 pg/mL [7.13–21.57 pg/mL]; *p* = 0.015; Figure 4b).

**Figure 4:**
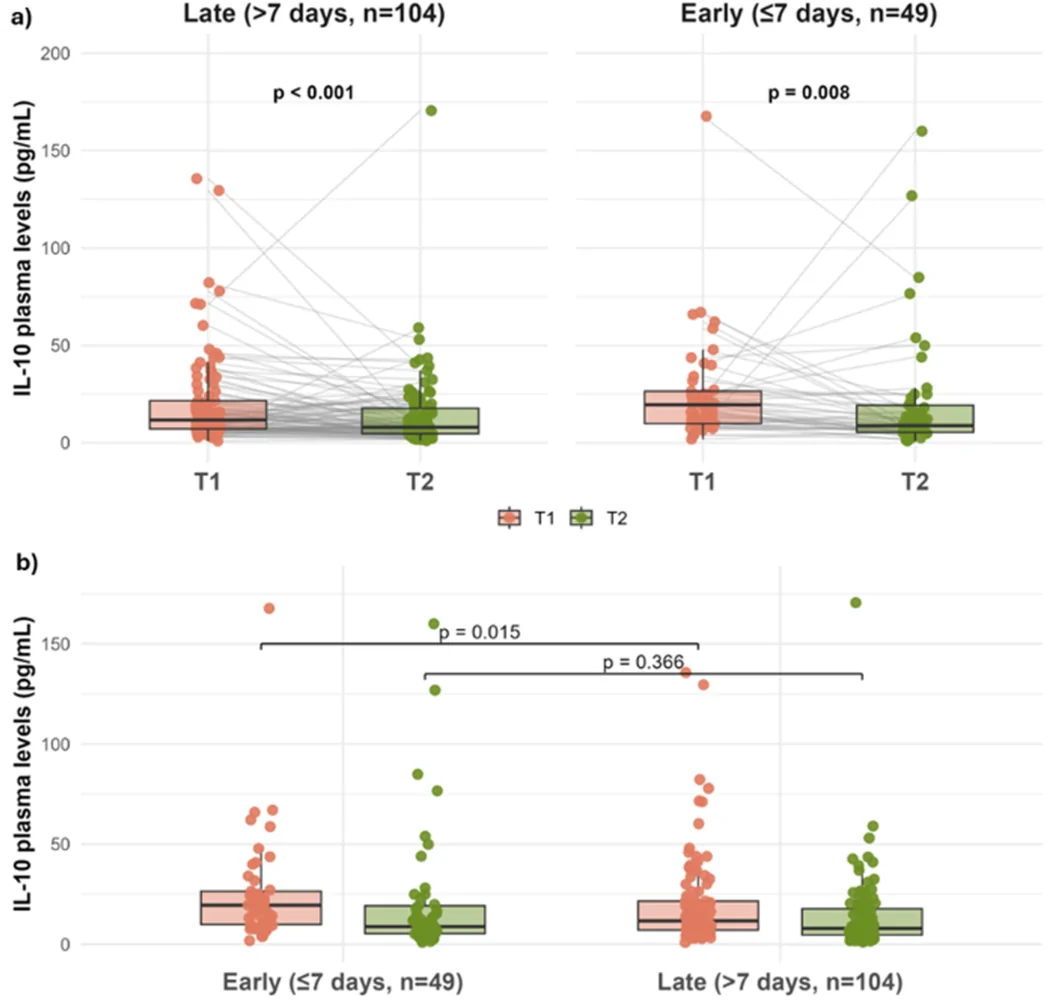
Differences in IL‐10 plasma levels based on the timing of corticosteroid initiation. (a) Differences in IL‐10 plasma levels at T1 and T2 depending on when corticosteroids are started, assessed with the Wilcoxon signed‐rank test. (b) Differences in IL‐10 plasma levels at each time point between the corticosteroid timing groups, evaluated with the Mann–Whitney *U*‐test. Early group: patients receiving corticosteroids within ≤ 7 days of symptom onset; Late group: patients receiving corticosteroids more than 7 days after symptom onset; T1: IL‐10 plasma levels at the start of corticosteroid treatment; T2: IL‐10 plasma levels after an average of 8 days of corticosteroid treatment.

The stratification by discharge outcome (survivors *n* = 107 and non‐survivors *n* = 46) revealed distinct patterns. Consistent with our previous results, IL‐10 plasma levels decreased in both timing groups among survivors (early, *p* < 0.001; late, *p* < 0.001); however, non‐survivors showed no significant T1–T2 variation in either the early or late groups (Figure 5).

**Figure 5:**
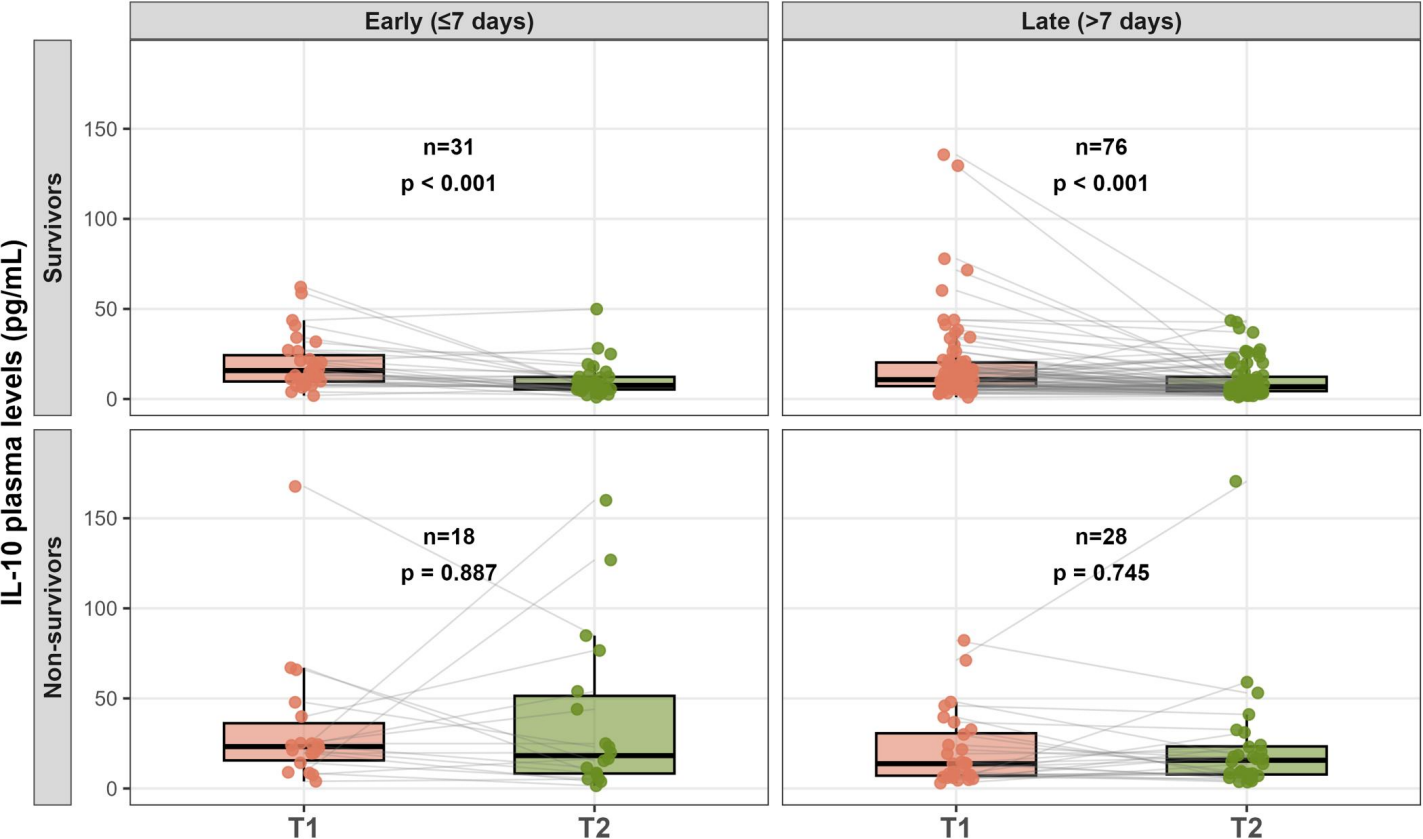
Differences in IL‐10 plasma levels by the time of corticosteroid initiation and in‐hospital mortality. Early: patients receiving corticosteroids within ≤ 7 days of symptom onset; Late: patients receiving corticosteroids > 7 days after symptom onset; T1: IL‐10 plasma levels at the onset of corticosteroid treatment; T2: IL‐10 plasma levels after a mean of 8 days of corticosteroid treatment. Wilcoxon signed‐rank test.

A second analysis was performed to investigate the effect of corticosteroid type on IL‐10 plasma levels. Based on the administered regimen, patients were classified as Dex if they received only dexamethasone during hospitalization (*n* = 114), and Dex + Others CS if they received additional corticosteroids (systemic or inhaled) in combination with at least one dose of dexamethasone (*n* = 46). The Wilcoxon signed‐rank test showed that IL‐10 plasma concentrations significantly decreased from T1 to T2 in the Dex group (*p* < 0.001), while no significant change was observed in the IL‐10 levels in the Dex + Others CS group (Figure S4a). Additionally, plasma levels at each time point (T1 and T2) did not differ significantly between the two corticosteroid treatment groups (Figure S4b). We also evaluated whether inhaled corticosteroids modify IL‐10 plasma levels, but there was no significant difference in IL‐10 plasma levels between patients receiving systemic treatment and those receiving systemic + inhaled corticosteroids (data not shown).

## 4 Discussion

During the COVID‐19 pandemic, the lack of established treatment represented a major challenge for healthcare professionals. Based on previous experience, corticosteroids were among the options to control the exacerbated inflammatory response. Even the efficacy and safety of corticosteroid treatment were debated [9, 24, 25, 26]. Initiation timing, COVID‐19 severity, and corticosteroid dosage, among other factors, were crucial determinants of the anti‐inflammatory treatment's efficacy. The inter‐individual variability in the corticosteroid treatment has also been widely described [12, 27, 28, 29, 30]. Here, we reported the association of the *IL10* rs1554286 and rs1800872 variants with the need for IMV in patients with severe COVID‐19 treated with corticosteroids. Additionally, we reported, for the first time, variation in IL‐10 plasma levels at two time points during corticosteroid treatment among patients with COVID‐19 and their relation to clinical outcome.

Several studies have evaluated the association between IL10 genetic variants and clinical outcomes in COVID‐19 across diverse populations. Most of these studies have focused on rs1800872 [31, 32, 33]. The association between rs1800872 and COVID‐19 severity and mortality has been controversial [33, 34, 35], and a meta‐analysis did not find an association between this variant and COVID‐19 mortality [34]. However, a previous study involving patients from Mexico reported an association of the polymorphism with the risk of severe COVID‐19 [35]. Conversely, we found an association between rs1554286 and the need for IMV, a measure of COVID‐19 severity, among hospitalized patients treated with corticosteroids. To the best of our knowledge, this variant has not been studied with respect to COVID‐19 mortality or severity risk; however, it has been proposed as an inflammation‐related genetic variant influencing susceptibility and vulnerability to coronavirus, respiratory syncytial virus, and influenza [36]. rs1554286 is an intronic variant related to the IL‐10 plasma levels in patients with ischemic stroke [37]. The exact impact of the polymorphism in the IL‐10 protein has not been described, but we observed it in LD with rs1800872, which is in the proximal promoter region and influences IL‐10 expression [38].

Regarding the IL‐10 plasma levels, several studies have demonstrated their relevance in predicting mortality and severity in COVID‐19 [39, 40, 41]; nevertheless, only one study has evaluated IL‐10 concentration in the context of response to corticosteroids, which reports an association between increased IL‐10 levels, measured only at the time of hospital admission, and resistance to treatment [14]. Our study highlights differences in IL‐10 levels measured at baseline and after an average of 8 days of corticosteroid treatment (T1 and T2), showing that persistent levels at T2 are associated with increased mortality and the need for mechanical ventilation in patients with COVID‐19. Other authors have also suggested that decreased IL‐10 levels are associated with clinical resolution, as we observed in this study [42, 43]. However, most of them recorded only a single IL‐10 serum measurement, limiting the ability to observe its dynamics during infection. In contrast, our two IL‐10 measurements enabled us to track this interleukin's behavior after corticosteroid treatment, and the results indicate that a systemic inflammatory response is maintained, which negatively affects disease prognosis. Whether these increased IL‐10 plasma levels are cause or consequence of the clinical outcome is not clear; however, this phenomenon has also been associated with mortality risk in other non‐respiratory diseases, such as patients with AIDS with disseminated Kaposi sarcoma [44], multiple myeloma [45], traumatic brain injury [46], and acute coronary syndrome [47]. Moreover, in the context of sepsis, sustained or mismatched IL‐10 plasma levels have been reported to impair antigen presentation and antimicrobial effector functions, contributing to sepsis, secondary infections, and poor outcomes [48], which could explain the association with mortality and IMV requirement in the studied patients with COVID‐19. In the context of hyperinflammation, sustained IL‐10 plasma levels could be correlated with tissue damage due to the uncontrolled immune response [42].

On the other hand, the upregulation of IL‐10 can be expected due to corticosteroid administration. Previous in vitro studies have reported enhanced IL‐10 production due to dexamethasone [49] or methylprednisolone [50] effects on T lymphocytes and macrophages, respectively. Although the deleterious effects of increased IL‐10 plasma levels remain controversial, in animal models, it has been reported that IL‐10's effects are context‐dependent, with positive and negative effects depending on the timing of administration and the severity of the infection in the context of the compensatory anti‐inflammatory response syndrome (CARS) [51, 52]. However, we did not observe a direct effect of corticosteroid administration timing on clinical outcome in patients with COVID‐19.

In the context of COVID‐19, another proposed explanation for the relation of the IL‐10 levels with poor outcomes included: the paradoxical effect of IL‐10 to act as a pro‐inflammatory and immunostimulatory molecule under particular scenarios; or the potential escape of the activated immune cells from IL‐10's anti‐inflammatory action under other inflammatory conditions [53]. However, we did not observe that IL‐10 levels at T2 were related to comorbidities, coinfections, or other factors beyond the clinical outcome. Further studies are warranted to elucidate the mechanisms underlying the increase in IL‐10 production and its relationship to poor clinical outcomes in COVID‐19 and other diseases characterized by dysregulation of the inflammatory response.

Our study is not exempt from limitations. The study of other cytokines and/or inflammation‐related biomarkers could help link corticosteroid effects on IL‐10 dynamics to clinical outcomes. The retrospective nature of the study affects the inclusion criteria for patients, particularly in the subgroup for IL‐10 plasma levels determination; however, its effect on clinical outcomes was observed when the group was classified accordingly. In addition, studying a group of patients with moderate‐to‐severe disease who did not receive corticosteroids could provide insights into the evolution of IL‐10 levels over the natural course of the disease. Further studies are necessary to clarify how host genetic variation and cytokine profiles modulate the inflammatory response to corticosteroids, and to explain the attenuated or heterogeneous treatment response seen in some patients with COVID‐19.

## Funding

The authors have nothing to report.

## Ethics Statement

The study was conducted in accordance with the Helsinki Declaration and received approval from the Local Committees for Research, Biosecurity, and Ethics in Research (approval code B03‐24).

## Consent

Written informed consent was obtained from each participant after they were invited and informed of the study's purpose and scope.

## Conflicts of Interest

The authors declare no conflicts of interest.

## Supporting information


Supporting File

## Data Availability

Data will be made available upon a reasonable request.
