# Supplementary material for: Plasma Interleukin‐10 Levels and IL10 Genetic Variants Are Associated With Clinical Outcomes in Patients With Severe COVID‐19 Treated With Corticosteroids
Source: J Med Virol. 2026 Oct 30;98(11):e71201. doi: 10.1002/jmv.71201 (PMC13721142; doi:10.1002/jmv.71201)
Supplement: Supplementary file 1 [file JMV-98-e71201-s001.pdf]

Supplementary Materials

Supplementary Table 1. Genetic association study of *IL10* variants with in-hospital mortality among patients with COVID-19 treated with corticosteroids.

IL10 variant Allele/Genotypes	Non-survivors (n=230[a])	Survivors (n=673[a])	*p*-value	Adjusted *p*-value [b]
rs3024505	**n=223**	**n=650**		
G	427 (0.957)	1229 (0.945)	0.321	-
A	19 (0.043)	71 (0.055)		
GG	204 (0.915)	579 (0.891)		
GA	19 (0.085)	71 (0.109)	0.310	0.215
AA	0	0		
rs3122605	**n=170**	**n=442**		
A	328 (0.963)	851 (0.965)	0.870	-
G	12 (0.037)	33 (0.035)		
AA	158 (0.929)	409 (0.925)		
AG	12 (0.071)	33 (0.075)	0.861	0.420
GG	0	0		
rs1554286	**n=219**	**n=652**		
G	223 (0.509)	647 (0.496)	0.640	0.506
A	215 (0.491)	657 (0.504)		
GG	54 (0.247)	156 (0.239)		
GA	115 (0.525)	335 (0.514)	0.310	0.422
AA	50 (0.228)	161 (0.247)		0.287
Dominant/recessive model				
GA+AA	165 (0.753)	496 (0.761)	0.830	0.321
GG	54 (0.247)	156 (0.239)		
AA	50 (0.228)	161 (0.247)	0.580	0.466
GG+GA	169 (0.772)	491 (0.753)		
rs1800872	**n=219**	**n=644**		
G	243 (0.555)	730 (0.567)	0.660	0.823
T	195 (0.445)	558 (0.433)		
GG	38 (0.173)	111 (0.172)		
GT	119 (0.543)	336 (0.522)	0.430	0.938
TT	62 (0.283)	197 (0.306)		0.575
Dominant/recessive model				
GT+TT	181 (0.826)	533 (0.828)	0.970	0.802
GG	38 (0.173)	111 (0.172)		
TT	62 (0.283)	197 (0.306)	0.520	0.551
GG+GT	157 (0.717)	447 (0.694)		

[a] Genotyping was performed based on sample availability; thus, the number genotyped is specified for each case. [b] Logistic regression model for the adjustment of covariates: age, body mass index, heart diseases, PaO_2/FiO_2 at admission, and coinfection.

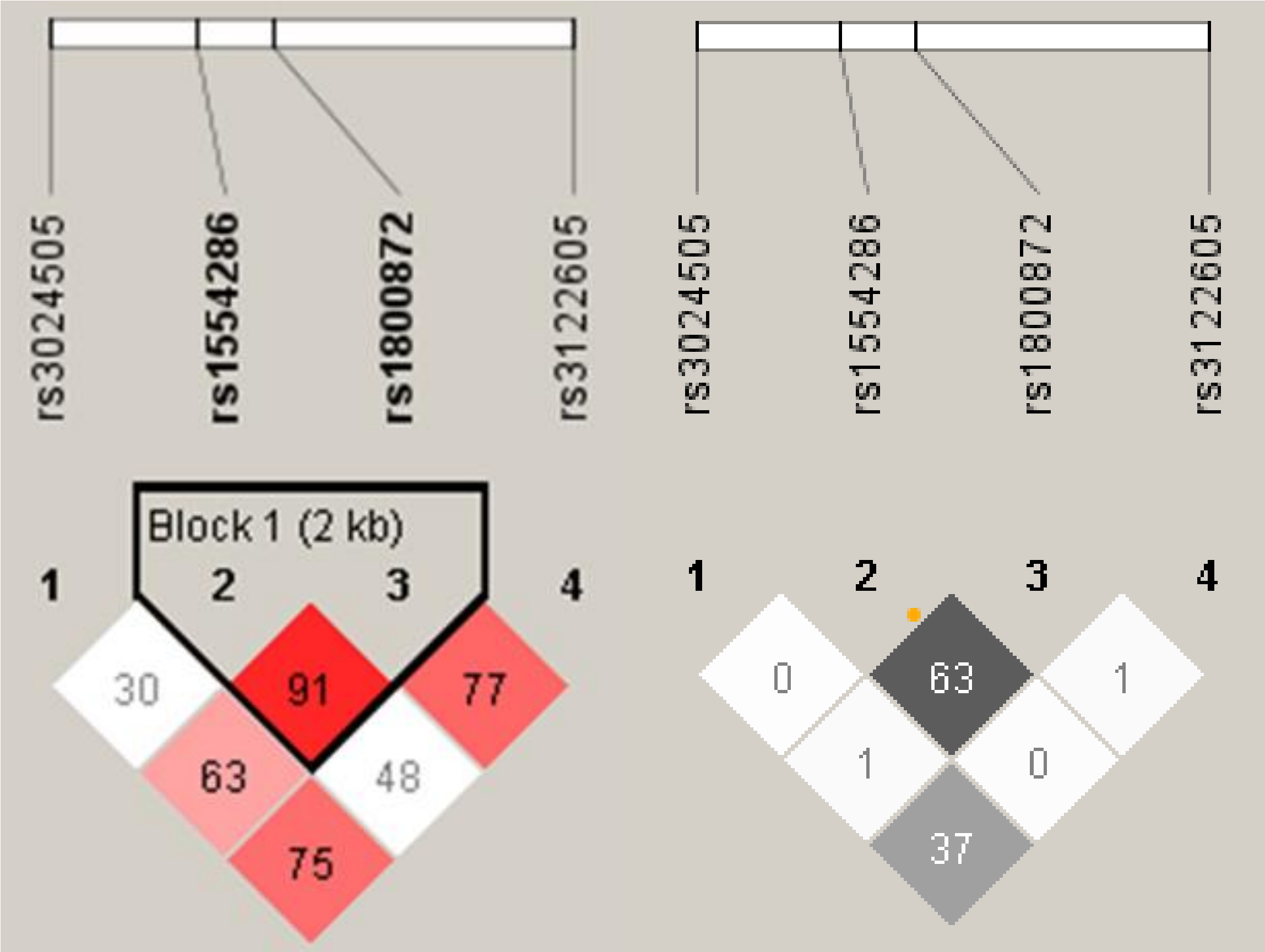

Supplementary Figure 1. Linkage disequilibrium plot of the *IL10* variants included in the genetic association study of invasive mechanical ventilation requirement in patients with COVID-19 receiving corticosteroid treatment. The numbers inside the diamonds indicate the D' values (left) and r^2 values (right). The intensity of red/dark color is proportional to the linkage disequilibrium between variants. The analysis was performed in Haploview.

Supplementary Table 2. Analysis of multicollinearity showing the Variance Inflation Factor (VIF) and tolerance levels for model covariates.

Covariable	VIF	Tolerance
Age	1.107	0.904
BMI	1.115	0.897
Heart disease	1.000	1.000
PaO_2/FiO_2	1.011	0.989
Coinfection	1.008	0.992

VIF, Variance Inflation Factor; BMI, Body Mass Index; PaO_2/FiO_2, ratio of arterial oxygen partial pressure to fractional inspired oxygen. Note: VIF values < 3.0 and tolerance levels > 0.10 indicate the absence of significant multicollinearity among the predictors.

Supplementary Table 3. Haplotype association study of *IL10* variants with the requirement for invasive mechanical ventilation in patients with COVID-19 receiving corticosteroid treatment.

Haplotypes rs1554286/ rs1800872	IMV	Non-IMV	*p*-value
GG	0.471	0.519	0.060
AT	0.418	0.390	0.264
AG	0.090	0.075	0.304
GT	0.021	0.016	0.449

Frequencies of the haplotypes in comparison groups. IMV, invasive mechanical ventilation. Linkage disequilibrium analysis performed in Haploview.

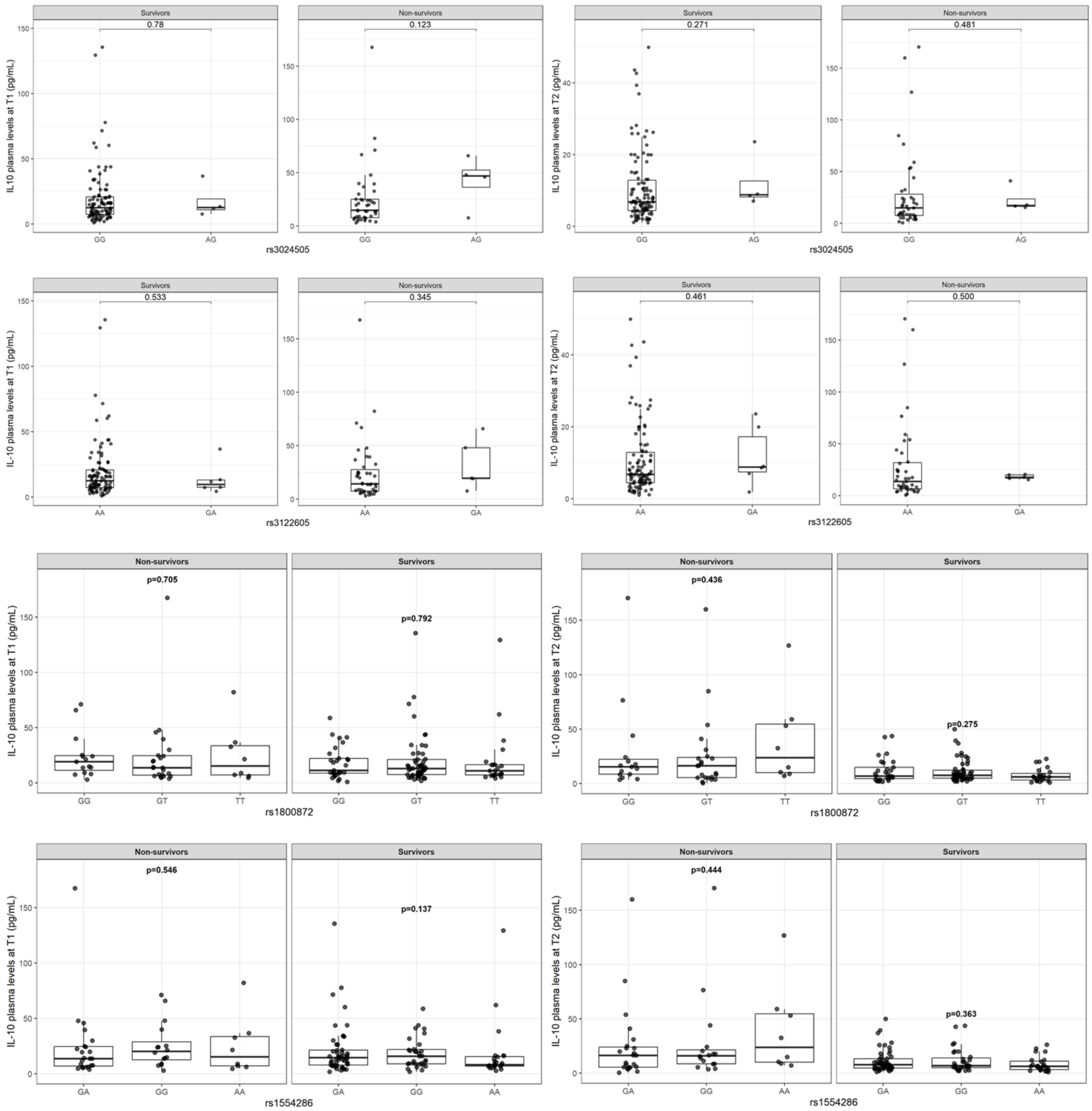

Supplementary Figure 2. Effect of the *IL10* rs3024505, rs3122605, rs1554286, and rs1800872 variants on IL-10 plasma levels at two time points during corticosteroid treatment in patients with severe COVID-19, classified by in-hospital mortality. Mann-Whitney U test or Kruskal-Wallis test, with multiple comparisons by the Dunn-Bonferroni method.

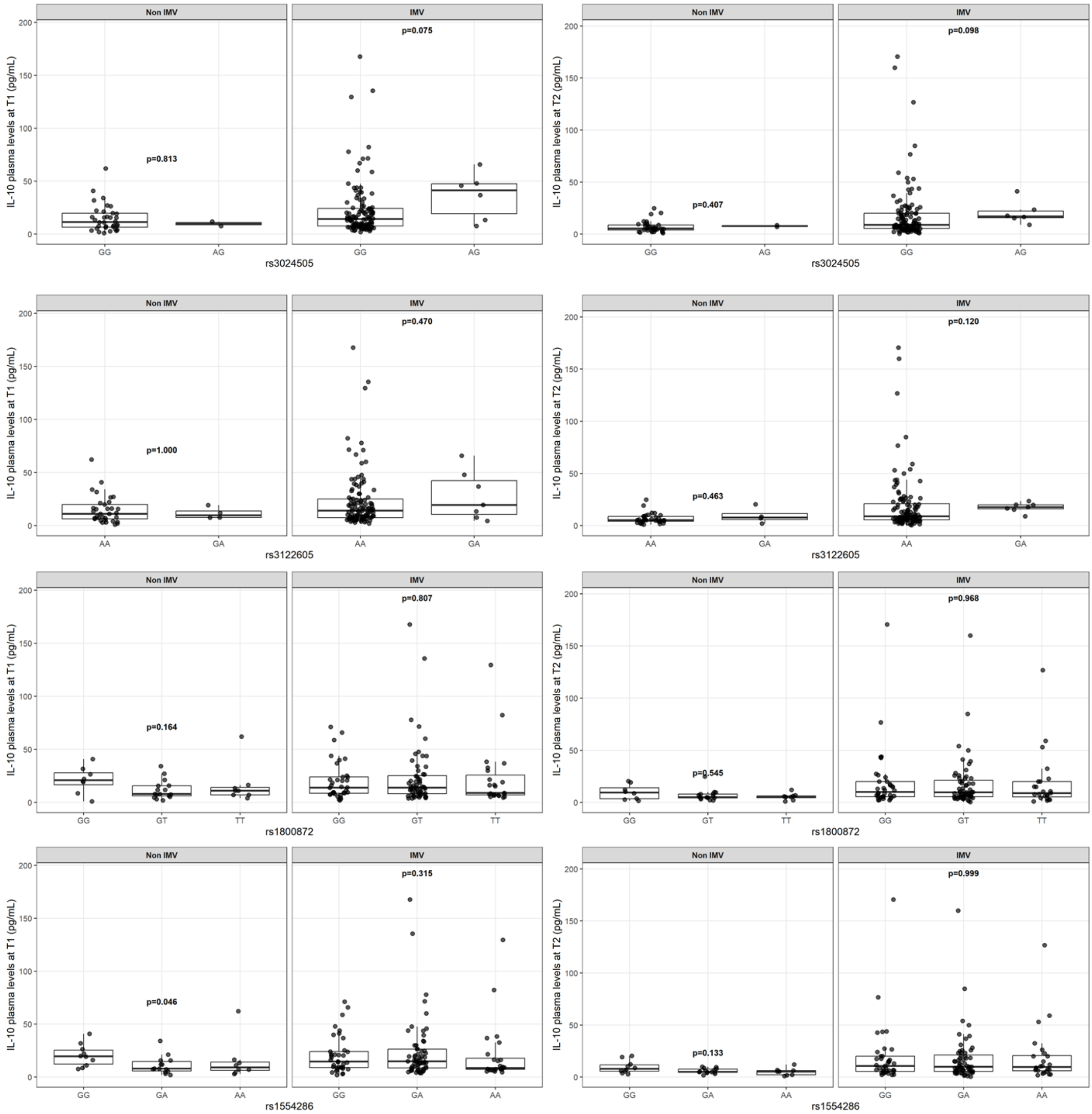

Supplementary Figure 3. Effect of the *IL10* rs3024505, rs3122605, rs1554286, and rs1800872 variants on IL-10 plasma levels at two time points during corticosteroid treatment in patients with severe COVID-19, classified by in-hospital mortality. Mann-Whitney U test or Kruskal-Wallis test, with multiple comparisons by the Dunn-Bonferroni method.

Supplementary Table 4. Clinical and demographic data for patients included in the analysis of IL-10 plasma levels (n=160) and the remaining patients (n=784) from the entire cohort (n=944).

Variable	IL-10 subgroup (n=160)	Rest of cohort (n=784)	*p value*
**Demographic**			
Age, years	60 [51.75 - 68.25]	57.00 [46 - 68]	**0.032**
BMI, kg/m^2	28.79 [25.77 - 33.45]	28.63 [25.59 - 32.53]	0.473
Sex, male n (%)	110 (68.7)	506 (64.5)	0.318
**Comorbidities n (%)**			
Tobacco-smoking	48 (30)	244 (31.1)	0.779
T2DM	51 (31.8)	213 (29.4)	0.246
SAH	64 (40)	268 (34.1)	0.173
CRD	8 (5)	63 (8)	0.248
Heart disease	7 (4.3)	28 (3.5)	0.645
**Clinical data at admission**			
Days since symptoms onset	8.5 [7-12]	10 [7-13]	**0.012**
PaO_2/FiO_2	163.62 [131-202.25]	157 [119.5-200]	0.118
COVID-19 vaccination n (%)	14 (35.9)	155 (19.7)	0.127
**Clinical outcomes**			
Coinfection n (%)	62 (38.7)	315 (40.17)	0.245
Hospital stay, days	20 [14-27]	16 [10-28]	**0.003**
IMV n (%)	123 (76.8)	530 (67.6)	**0.023**
Mortality n (%)	48 (30)	187 (23.8)	0.163

Continuous data are presented as median [interquartile range (Q1-Q3)]. Categorical data are presented in absolute numbers (%). Exact Fisher's and Mann-Whitney U tests were used for comparisons. CRD, chronic respiratory diseases; BMI, body mass index; IMV, invasive mechanical ventilation; SAH, systemic arterial hypertension; T2DM, type 2 diabetes mellitus. Text in bold denotes a significant *p*-value

Supplementary Table 5. Relationship between clinical and demographic variables and IL-10 plasma levels at two time points during corticosteroid treatment in patients with COVID-19.

Variable	T1 IL-10 plasma levels (pg/mL)		*p*-value	T2 IL-10 plasma levels (pg/mL)		***p-value***
	**Yes**	**No**		**Yes**	**No**	
SAH	16.4 (8.4–35.2)	12.00 (7.3–20.1)	**0.027**	8.7 (5.4–21.0)	8.1 (4.8–15.1)	0.169
Sex, male	13.5 (7.4–22.3)	14.6 (7.7–24.9)	0.594	9.2 (5.2–20.0)	7.1 (4.9–12.9)	0.155
Smoking	15.3 (8.0–25.3)	13.0 (7.4–22.6)	0.407	6.9 (4.4–14.0)	8.9 (5.3–19.9)	0.144
T2DM	14.1 (7.2–25.8)	13.6 (7.5–22.1)	0.943	8.3 (5.6–20.2)	8.4 (4.5–16.4)	0.313
CRD	10.2 (7.7–30.1)	13.8 (7.5–22.9)	0.966	7.4 (4.4–10.6)	8.5 (5.1–18.7)	0.316
Heart disease	36.8 (11.4–43.9)	13.5 (7.5–22.1)	0.157	23.1 (7.1–27.8)	8.2 (4.8–17.6)	0.251
Coinfection	14.9 (9.1–32.4)	14.1 (7.8–24.0)	0.359	10.4 (6.5–22.3)	9.6 (5.3–19.0)	0.299
COVID-19 vaccine	13.6 (7.6–27.6)	10.0 (7.5–21.5)	0.349	8.1 (6.1–8.9)	9.6 (6.3–19.9)	0.438

The IL-10 plasma levels are presented as median [interquartile range, Q1-Q3]. Mann-Whitney U test was used for comparisons. CRD, chronic respiratory diseases; SAH, systemic arterial hypertension; T1, IL-10 plasma levels at the start of corticosteroid treatment; T2, IL-10 plasma levels at a mean of 8 days into corticosteroid treatment; T2D, type 2 diabetes mellitus. Text in bold denotes a significant *p*-value

Supplementary Table 6. Correlation analysis of clinical and demographic variables with IL-10 plasma levels at two time points during corticosteroid treatment in patients with COVID-19.

Clinical and demographic variables	T1 IL-10 plasma levels (pg/mL)		T2 IL-10 plasma levels (pg/mL)	
	**ρ**	***p*-value**	**ρ**	***p*-value**
Age, years	0.201	**0.011**	0.129	0.104
BMI (kg/m^2)	-0.033	0.685	-0.151	0.061
Hospital stay days	0.015	0.855	0.256	**<0.001**
PaO_2/FiO_2 at admission	0.051	0.522	-0.142	0.074
Days since symptoms onset	-0.19	**0.016**	-0.039	0.629

The correlation analyses were performed with the Spearman rank correlation test. BMI, body mass index; T1, IL-10 plasma levels at the start of corticosteroid treatment; T2, IL-10 plasma levels at a mean of 8 days into corticosteroid treatment. Text in bold denotes a significant *p*-value.

Supplementary Table 7. Transition of patients categorized into tertiles of IL-10 plasma levels from T1 to T2.

T1 tertile (range)	T2 tertile (range)	n	Row %
0.93 pg/mL to < 8.71 pg/mL	0.38 pg/mL to < 5.88 pg/mL	24	45.3
	5.88 pg/mL to < 13.10 pg/mL	20	37.7
	≥ 13.10 pg/mL	9	17
8.71 pg/mL to < 19.85 pg/mL	0.38 pg/mL to < 5.88 pg/mL	20	37.7
	5.88 pg/mL to < 13.10 pg/mL	16	30.2
	≥ 13.10 pg/mL	17	32.1
≥ 19.85 pg/mL	0.38 pg/mL to < 5.88 pg/mL	9	16.7
	5.88 pg/mL to < 13.10 pg/mL	17	31.5
	≥ 13.10 pg/mL	28	51.9

T1, IL-10 plasma levels at the onset of corticosteroid treatment; T2, IL-10 plasma levels after a mean of 8 days of corticosteroid treatment. IL-10 plasma levels at T1 and T2 were categorized according to tertiles.

Supplementary Table 8. Transition of patients from each tertile of IL-10 plasma levels at T2 to the invasive mechanical ventilation requirement and in-hospital mortality.

T2 tertile (range)	IMV status	In-hospital morality	n	% within T2
0.38 pg/mL to < 5.88 pg/mL	Non-IMV	Survivors	19	35.8
	IMV	Survivors	24	45.3
	IMV	Non-survivors	10	18.9
5.88 pg/mL to < 13.10 pg/mL	Non-IMV	Survivors	15	28.3
	IMV	Survivors	27	50.9
	IMV	Non-survivors	11	20.8
≥ 13.10 pg/mL	Non-IMV	Survivors	2	3.7
	Non-IMV	Non-survivors	1	1.9
	IMV	Survivors	25	46.3
	IMV	Non-survivors	26	48.1

IMV, invasive mechanical ventilation; T1, at the onset of corticosteroid treatment; T2, after a mean of 8 days of corticosteroid treatment. IL-10 plasma levels at T1 and T2 were categorized according to tertiles.

Supplementary Table 9. Frequency of patients in each tertile of IL-10 plasma levels at T2, and their clinical outcomes according to invasive mechanical ventilation requirement and in-hospital mortality.

**T2 tertile (range)**	**IMV status**	**n**	**Row %**	**In-hospital mortality**	**n**	**Row %**
0.38 pg/mL to < 5.88 pg/mL	Non-IMV	19	35.8	Survivors	43	81.1
	IMV	34	64.2	Non-survivors	10	18.9
5.88 pg/mL to < 13.10 pg/mL	Non-IMV	15	28.3	Survivors	42	79.2
	IMV	38	71.7	Non-survivors	11	20.8
≥ 13.10 pg/mL	Non-IMV	3	5.6	Survivors	27	50
	IMV	51	94.4	Non-survivors	27	50

IMV, invasive mechanical ventilation; T1, at the onset of corticosteroid treatment; T2, after a mean of 8 days of corticosteroid treatment. IL-10 plasma levels at T1 and T2 were categorized according to tertiles.

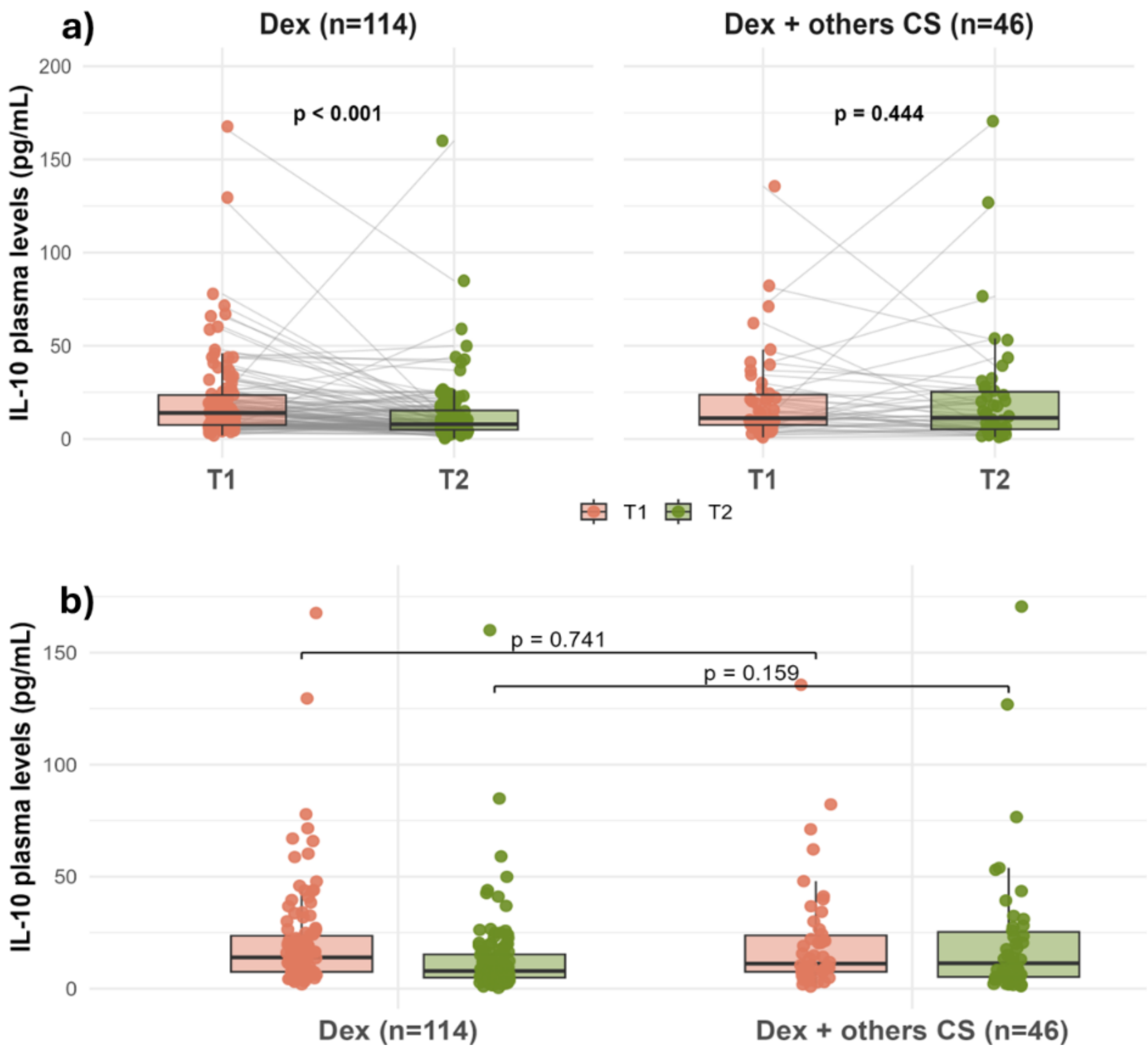

Supplementary Figure 4. Differences in IL-10 plasma levels based on corticosteroid treatment type. a) Differences in IL-10 plasma levels at T1 and T2 depending on corticosteroid treatment, using the Wilcoxon signed-rank test. **b)** Differences in IL-10 plasma levels at each time point between groups by corticosteroid treatment type, using the Mann-Whitney U test. Dex: patients receiving only dexamethasone during hospitalization; Dex + Others CS: patients receiving additional corticosteroids (systemic or inhaled) along with at least one dose of dexamethasone; T1: IL-10 plasma levels at the start of corticosteroid treatment; T2: IL-10 plasma levels after a mean of 8 days of corticosteroid treatment.
